# Assessment of Dextran Antigenicity of Intravenous Iron Preparations with Enzyme-Linked Immunosorbent Assay (ELISA)

**DOI:** 10.3390/ijms17071185

**Published:** 2016-07-21

**Authors:** Susann Neiser, Taija S. Koskenkorva, Katrin Schwarz, Maria Wilhelm, Susanna Burckhardt

**Affiliations:** 1 Chemical and Preclinical Research and Development, Vifor International Ltd., 9014 St. Gallen, Switzerland; susann@neiser.ch (S.N.); katrin.schwarz@viforpharma.com (K.S.); maria.wilhelm@viforpharma.com (M.W.); 2 Global Medical Affairs, Vifor Pharma Ltd., 8152 Glattbrugg, Switzerland; taija.koskenkorva@gmail.com

**Keywords:** anaphylaxis, antidextran, intravenous iron

## Abstract

Intravenous iron preparations are typically classified as non-dextran-based or dextran/dextran-based complexes. The carbohydrate shell for each of these preparations is unique and is key in determining the various physicochemical properties, the metabolic pathway, and the immunogenicity of the iron-carbohydrate complex. As intravenous dextran can cause severe, antibody-mediated dextran-induced anaphylactic reactions (DIAR), the purpose of this study was to explore the potential of various intravenous iron preparations, non-dextran-based or dextran/dextran-based, to induce these reactions. An IgG-isotype mouse monoclonal anti-dextran antibody (5E7H3) and an enzyme-linked immunosorbent assay (ELISA) were developed to investigate the dextran antigenicity of low molecular weight iron dextran, ferumoxytol, iron isomaltoside 1000, ferric gluconate, iron sucrose and ferric carboxymaltose, as well as isomaltoside 1000, the isolated carbohydrate component of iron isomaltoside 1000. Low molecular weight iron dextran, as well as dextran-based ferumoxytol and iron isomaltoside 1000, reacted with 5E7H3, whereas ferric carboxymaltose, iron sucrose, sodium ferric gluconate, and isolated isomaltoside 1000 did not. Consistent results were obtained with reverse single radial immunodiffusion assay. The results strongly support the hypothesis that, while the carbohydrate alone (isomaltoside 1000) does not form immune complexes with anti-dextran antibodies, iron isomaltoside 1000 complex reacts with anti-dextran antibodies by forming multivalent immune complexes. Moreover, non-dextran based preparations, such as iron sucrose and ferric carboxymaltose, do not react with anti-dextran antibodies. This assay allows to assess the theoretical possibility of a substance to induce antibody-mediated DIARs. Nevertheless, as this is only one possible mechanism that may cause a hypersensitivity reaction, a broader set of assays will be required to get an understanding of the mechanisms that may lead to intravenous iron-induced hypersensitivity reactions.

## 1. Introduction

Iron is an essential component of the body. However, when present in excess, iron is toxic [1] and has the potential to induce oxidative stress [2]. Thus, iron levels are under tight control: Iron uptake via the duodenum is strictly regulated, resulting in only small amounts of iron to be absorbed daily [3]. Intravenous (IV) iron therapy is used to treat iron deficiency (ID) and iron deficiency anemia (IDA), when there is a need for fast replenishment or when oral iron is ineffective or not tolerated [4]. Because IV administration bypasses the strictly regulated iron absorption in the gut, it is critical that IV iron preparations are engineered to deliver high doses of iron in a stable, non-reactive and non-toxic form.

All preparations for IV iron therapy are composed of carbohydrate-stabilized polynuclear iron(III)-oxyhydroxide/oxide nanoparticles formulated as colloidal solutions. Thus, they are non-biological complex drugs (NBCDs) [5,6]. The carbohydrate shell is unique for each preparation. In addition to stabilizing the iron core in a ligand-specific way, the shell is the key component regulating the stability, size, shape and surface charge of the iron-carbohydrate complex [2]. Thus, upon IV administration, the carbohydrate shell determines the metabolic pathway of the complexes, affecting their pharmacokinetics and pharmacodynamics, as well as their interaction with the innate immune system and, thus, side effects [2].

Current IV iron preparations on the market in Europe and/or in the US include iron sucrose (IS), ferric carboxymaltose (FCM), sodium ferric gluconate (SFG), iron isomaltoside 1000 (IIM), ferumoxytol (FMX), and low molecular weight iron dextran (LMWID). Depending on the type of the carbohydrate shell, these preparations can be classified as (a) non-dextran-based and (b) dextran/dextran-based complexes [7]. Non-dextran-based complexes exhibit a correlation between molecular weight distribution and complex stability, i.e., complexes with higher molecular weight are more stable and have lower labile iron content than complexes with lower molecular weight [8,9]. In contrast, dextran/dextran-based complexes are all very stable independent of their molecular weight [1,8,9].

A previous reverse single radial immunodiffusion assay demonstrated that LMWID, FMX and IIM reacted with an anti-dextran antibody, whereas IS, SFG and FCM did not [10]. However, as this methodology was criticized [11], a new monoclonal anti-dextran antibody (mouse IgG-isotype) and an enzyme-linked immunosorbent assay (ELISA) were developed [12]. As intravenous dextran can cause severe, antibody-mediated dextran-induced anaphylactic reactions (DIARs), the purpose of this study was to assess the overall possibility of a complex formation with anti-dextran antibodies of the different non-dextran-based or dextran/dextran-based IV iron preparations as well as of the isolated carbohydrate components. The results strongly support the hypothesis that, while the carbohydrate alone (isomaltoside 1000, IM1000) does not form immune complexes with anti-dextran antibodies, iron isomaltoside 1000 complex reacts with anti-dextran antibodies by forming multivalent immune complexes. Moreover, non-dextran based preparations, such as iron sucrose and ferric carboxymaltose, do not react with anti-dextran antibodies.

## 2. Results

In the newly developed ELISA assay, a positive result was defined as A_450_ ratio (sample/blank) ≥2.1. The results against the antigen used for antibody production (dextran 50,000) resulted in a titer of >1:81,000, i.e., 12.3 ng/mL gave a positive reaction.

Two separate ELISA experiments were carried out. In the first, the six different IV iron preparations were assessed and in the second, IIM was reassessed together with its carbohydrate component IM1000. Positive reactions against 5E7H3 were observed for FMX, IIM, LMWID, and dextran 5000 (positive control) (Table 1 and Table 2). The endpoint titers against LMWID, FMX and IIM were >1:81,000, 1:27,000 and 1:3000–1:8000, respectively. FCM, IS, SFG, dextran 1000 (negative control) (Table 1) and IM1000 (Table 2) did not react with 5E7H3. Despite the numerical differences, the results of IIM and dextran 5000 (positive control) were comparable between the two experiments, i.e., the endpoint titers of the positive control and IIM were in a similar range.

**Figure 1:**
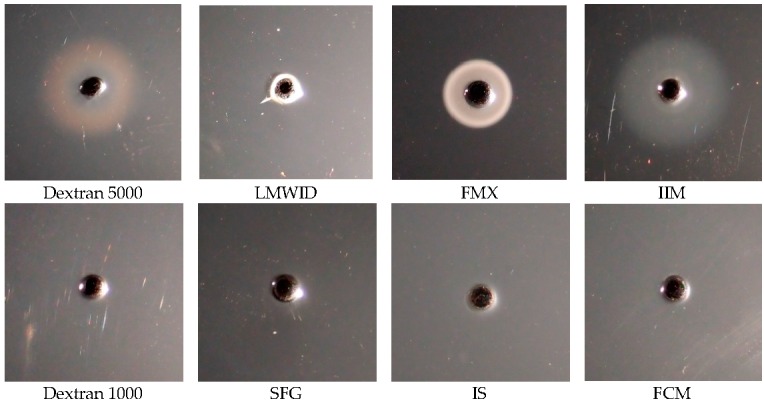
Reactivity of different IV iron preparations with 5E7H3 by reverse single radial immunodiffusion. Positive antigen/antibody reaction, indicated by circular turbidity around the well, is observed in the upper row for the positive control (dextran 5000) and the dextran/dextran-based preparations (LMWID, FMX, and IIM). In the lower row, the negative control (dextran 1000) as well as the non-dextran-based preparations (SFG, IS, and FCM) do not show any reaction. FCM, ferric carboxymaltose; FMX, ferumoxytol; IIM, iron isomaltoside 1000; IS, iron sucrose; LMWID, low molecular weight iron dextran; SFG, sodium ferric gluconate.

In addition to ELISA, reverse single radial immunodiffusion assay was performed with 5E7H3 (Figure 1). As expected, a distinct precipitation ring, indicative of a positive reaction, was observed with dextran 5000 (positive control), whereas dextran 1000 (negative control) did not form a precipitate with the antibody. The results of the experiments with the IV iron preparations were in agreement with the ELISA results (Table 3) and with our previous results obtained by using a different antibody [10]. Positive antigen/antibody reaction was observed with the dextran/dextran-based preparations LMWID, FMX, and IIM, whereas the non-dextran-based preparations SFG, IS, and FCM did not show any reaction. Although the intensities of the observed precipitation rings differed between the preparations, they were all clearly visible and stronger than or comparable to that of the positive control. Thus, the new data confirm the validity of this method.

## 3. Discussion

Injection of any IV drug can cause a hypersensitivity reaction (HSR). The prevalence of HSRs varies greatly between drugs and spans over a very broad range (0.001%–70%) among all patients treated [13]. The risk for an HSR in patients given IV iron is generally very low [13]. However, the rates have been shown to be product-dependent and to be lower for non-dextran-based complexes [14,15]. In particular, higher rates and generally more severe reactions, including deaths, have previously been reported with high molecular weight iron dextran complexes, most of which are no longer marketed [16,17]. To date, the exact mechanisms of IV iron-induced HSRs have not been elucidated [13]. The majority are considered non-IgE-mediated [13,18,19,20], and several lines of indirect evidence suggest that C-activation-related pseudoallergy (CARPA) may play a causal role in IV iron-induced HSRs [13].

It has long been known that IV dextran, commonly used as a plasma expander, can cause severe, IgG-mediated type III immune complex anaphylaxis [21,22,23] also known as severe, antibody-mediated DIARs. Therefore, the development of some of the new IV iron preparations focused on compounds, which are unlikely to have antigenic potential, e.g., by using carbohydrates free of dextran and its derivatives [24]. Alternatively, to reduce the antigenicity of dextran-based IV iron complexes, attempts have been made to use lower molecular weight or chemically modified dextrans [8,25]. However, as demonstrated here, modification of dextran in a way that prevents it from reacting with anti-dextran antibodies is challenging.

Due to the low frequency of antibody-mediated DIAR, a risk assessment for dextran-antigenicity of a new product is almost impossible in the setting of regulatory clinical trials [26]. Furthermore, patients with known hypersensitivity to iron dextran [27,28,29], IV iron products [25,30,31], or multiple drug hypersensitivity are often excluded from clinical studies [32]. In this study, the reactivity of six IV iron preparations against an anti-dextran antibody (5E7H3) was studied in vitro with an ELISA. Not surprisingly, LMWID resulted in the highest reactivity with 5E7H3, followed by FMX, IIM and dextran 5000 (positive control). The dextran-reactive antibody 5E7H3 was generated against dextran 50,000, which, having high molecular weight, contains a large number of the units of five to six α-(1→6)-linked glucose molecules necessary for recognition by an anti-dextran antibody [33]. The extent of binding between 5E7H3 and the tested IV iron preparations correlates with the number of antibody-recognizable units, allowing quantitative assessment of the dextran antigenicity of the studied preparations.

The reaction of LMWID with 5E7H3 was still strong at a dilution of 1:81,000, corresponding to the reactivity of dextran 50,000, which was used to generate the antibody. The carbohydrate component of LMWID is a dextran with a *M*_W_ of about 5000 Da [8] and, thus, around 25 glucose units. It has been shown previously that low molecular weight iron dextran complexes have a greater capacity to react with anti-dextran antibodies than the low molecular weight dextran carbohydrate component alone [34], most likely due to the repetitive and organized array of antigens on the complex, which favors the formation of immune complexes [35,36]. Indeed, the dextran antigenicity of LMWID complex was much higher than that of dextran 5000, which was used as the positive control (Table 1).

**Figure 2:**
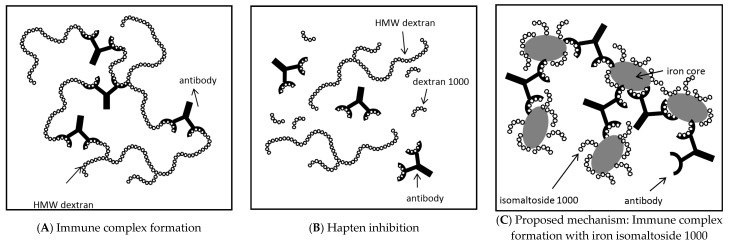
Schematic illustration of immune complex formation and inhibition. (**A**) Immune complex formation with high molecular weight (HMW) dextran and anti-dextran antibody. A large insoluble complex is generated, which may trigger DIARs; (**B**) Inhibition of immune complex formation by dextran 1000, i.e., hapten prophylaxis. Dextran 1000 binds to the anti-dextran antibody without formation of an immune complex and thus does not induce any immune response. The anti-dextran antibody is no longer available for binding to the HMW dextran, which thus is less likely to induce DIARs; (**C**) Proposed mechanism of the observed in vitro multivalent immune complex formation with iron isomaltoside 1000 (IIM) and anti-dextran antibody. In IIM, a number of isomaltoside 1000 (reduced dextran 1000) units are attached to the polynuclear iron core. The presented in vitro results suggest that IIM can act as a polyvalent higher molecular weight dextran and lead to immune complex formation. As in (**A**), a large insoluble complex is formed, which may trigger DIARs. (**A**,**B**) Based on Richter et al. [40].

The carbohydrate component of IIM, isomaltoside 1000, is a linear reduced dextran 1000 [37,38], i.e., it consists of approximately five α-(1→6)-linked glucose units [9]. Similarly to dextran 1000, it has a *M*_W_ of about 1000 Da [9,37,38]. As demonstrated for dextran 1000 (Figure 2B) [39], isomaltoside 1000 has been suggested to act as a monovalent antigen (i.e., hapten) by blocking the antibody binding sites without the formation of immune complexes [8,38]. Our in vitro tests confirm this hypothesis, as the carbohydrate component IM1000 did not show a positive reaction in the ELISA with 5E7H3. However, in contrast, IIM complex reacted with 5E7H3 and gave positive ELISA results. This can be explained by the fact that the IIM complex contains a number of isomaltoside 1000 ligands bound to the polynuclear iron core. Since each of the isomaltoside 1000 molecules can react with an anti-dextran antibody, a multivalent immune complex may be formed (Figure 2C) [10], as suggested previously for iron dextran 1000 complexes [4].

Additionally, FMX reacted strongly with 5E7H3. The FMX carbohydrate, polyglucose sorbitol carboxymethylether (PSC), is a carboxymethylated and reduced dextran, composed of 20–22 glucose units [9]. PSC is also added to the FMX solution as an excipient [41,42]. Its carboxymethylation degree is approximately 0.2 [9]. Interestingly, Richter et al. [43] have shown that carboxymethylated dextrans with a substitution degree ≤0.15 react with anti-dextran antibodies, whereas a carboxymethylated dextran with a substitution degree of 0.5 does not. Moreover, although pre-clinical tests did not suggest antigenicity [44], a monoclonal anti-dextran antibody was effectively used to localize FMX in histologic sections of synovium in rats with experimental immune complex arthritis [45]. Thus, these observations suggest that carboxymethylation degree of 0.2 for PSC, corresponding to one carboxymethylated unit for every 4–5 glucose units, is not enough to prevent in vitro reaction of FMX with anti-dextran antibodies. It is also possible that, similarly as for LMWID [34] and despite the carboxymethylation, the reactivity of FMX is relatively high due to the increase in molecular size resulting from the formation of the iron-PSC complex. Thus, more of the antigenic determinant may be available to react with the antibody.

## 4. Materials and Methods

IV iron preparations were obtained from a pharmacy or directly from the manufacturer: 30 mg Fe/mL FMX (Feraheme^®^, AMAG Pharmaceuticals, Inc., Lexington, MA, USA), 100 mg Fe/mL IIM (MonoFer^®^, Pharmacosmos A/S, Holbaek, Denmark), 50 mg Fe/mL FCM (Ferinject^®^, Vifor (International) Inc., St. Gallen, Switzerland), 20 mg Fe/mL IS (Venofer^®^, Vifor (International) Inc.), 50 mg Fe/mL LMWID (Cosmofer^®^, TEVA GmbH, Radebeul, Germany), and 12.5 mg Fe/mL SFG (Ferrlecit^®^, Watson Laboratories, Weston, FL, USA). The used lot numbers are indicated for each method separately. The carbohydrate component IM1000 was isolated from IIM solution, as described previously [9].

The dextran standards were dextran 1000 (Fluka, product no. 31416, lot BCBD4347V-02-002, Sigma-Aldrich, Buchs, Switzerland) with weight average molecular weight (*M*_W_) of 1270 Da (negative control), dextran 5000 (lot 00309, Serumwerke Bernburg AG, Bernburg, Germany) with *M*_W_ of 4500 Da (positive control), dextran 5000 (lot F201, Serumwerke Bernburg AG) with *M*_W_ of 5320 Da (positive control), and dextran 50,000 (Fluka, product no. 00891, lot BCBC7090, Sigma Aldrich, Buchs, Switzerland) with a *M*_W_ of 48,600 Da.

The anti-dextran antibody 5E7H3 was developed by GenScript (Piscalaway, NJ, USA) by immunizing five BALB/c mice with a conjugate of dextran 50,000 and Keyhole Limpet Hemocyanin. The monoclonal antibody (mouse IgG1-isotype) was produced in hybridoma cells by standard techniques.

### 4.1. Reverse Single Radial Immunodiffusion Assay

The new anti-dextran antibody 5E7H3 (GenScript) was used for the reverse single radial immunodiffusion assay, which was carried out with 1 mg/mL 5E7H3 following the procedure described previously [10]. Agar sample plates were prepared by mixing 8 mL supporting agar solution with 2 mL of the IV iron preparation solutions or control solutions. The IV iron preparation solutions were obtained by diluting them with water to an iron concentration of 40 μg/mL. Dextran 1000 (negative control) was tested in concentrations of 3 and 66 μg dextran/mL, in 0.9% (m/V) aqueous NaCl solution. Dextran 5000 (positive control) was tested at concentrations of 0.5, 1, 2, 3, and 5 μg/mL.

### 4.2. Indirect Enzyme-Linked Immunosorbent Assay (ELISA)

The six studied IV iron preparations and IM1000 were provided as blinded samples and the method development and testing of the samples was done by GenScript.

A microtiter plate was coated by adding 100 µL of the antigen-containing solutions in concentrations of 1 μg iron/mL in PBS for FMX, IIM, FCM, IS, SFG, and LMWID solutions, and 1 µg/mL in PBS for IM1000 and the dextran standards. Plates were covered with adhesive plastic and incubated at 37 °C for 2 h or at 4 °C overnight. After removing the antigen coating solution, wells were first washed three times with 250 µL washing buffer (PBS with 0.05% Tween 20), after which 200 µL blocking buffer (1% BSA in PBS) was added and incubated at 37 °C for 1 h to block the non-specific binding sites in the coated wells. The primary antibody 5E7H3 was diluted with the blocking buffer and 100 µL of the dilutions were added to the wells and incubated at 37 °C for 1 h. After the diluted antibody solution was removed the wells were washed three times with 250 µL washing buffer. The secondary antibody, horseradish-peroxidase-labeled conjugated affinity purified anti-mouse IgG (H & L, cat. No. 610-1302, Rockland Immonochemicals Inc., Limerick, PA, USA), was diluted to 0.2 µg/mL with blocking buffer and 100 µL of the diluted antibodies was added to each well and incubated at 37 °C for 30 min. The plate was washed five times with 250 µL washing buffer after which 100 µL of 3,3′,5,5′-tetramethylbenzidine reagent (TMB, cat. No. M00078, GenScript) was added to each well to react with the conjugates. After color development (10–15 min), 100 µL stop buffer (8.3 mL 12 mol/L HCl + 91.7 mL ddH_2_O) was added to the wells and the absorbance was recorded at 450 nm (A_450_).

## 5. Conclusions

Two different in vitro assays showed that, in addition to LMWID, dextran-based IV iron preparations FMX and IIM react with anti-dextran antibody, suggesting that antibody-mediated DIARs can occur in vivo. This study also confirms that, in contrast to the isolated ligand (IM1000), the IIM complex reacts with anti-dextran antibodies, possibly due to the high number of isomaltoside 1000 ligands on the surface of the polynuclear iron core, resulting in the formation of a multivalent immune complex. Thus, the non-immunogenicity of the carbohydrate does not guarantee that the iron-carbohydrate complex is non-immunogenic. Clearly, as confirmed by our in vitro tests, non-dextran-based IV iron preparations FCM, IS and SFG do not carry the risk of antibody-mediated DIAR.

Assays, such as those used in this work, are an approach to assess the theoretical possibility of a substance to induce antibody-mediated DIARs. Nevertheless, the likelihood that these preparations provoke antibody-mediated DIARs in vivo, and thus the clinical relevance of these results, cannot be assessed based on these assays alone. To date, no antibody-mediated DIARs have been reported for IIM. However, FDA raised safety concerns [26] shortly after the approval of FMX in June 2009, and a case of a possibly dextran-mediated anaphylaxis has been reported [42]. Finally, as DIARs are only one of the possible mechanisms that may trigger a hypersensitivity reaction, a broader set of assays will be required to unravel the mechanisms that may induce IV iron-induced hypersensitivity reactions.

## Figures and Tables

**Table 1:** ELISA results, A_450_ ratios (sample/blank), for the reactivity of different IV iron preparations with 5E7H3 antibody ^a^.

Antibody Dilution	1:1000 ^b^	1:3000	1:9000	1:27,000	1:81,000
LMWID	**17.3**	**15.8**	**14.0**	**11.7**	**8.0** ^c^
FMX	**12.7**	**8.7**	**4.4**	**2.2** ^c^	1.5
IIM	**3.9**	**2.4** ^c^	1.6	1.1	1.3
FCM	1.2	1.1	1.4	1.9	1.1
IS	0.8	1.2	1.1	1.6	0.8
SFG	0.7	0.8	2.0	1.3	1.3
Dextran 50,000	**35.0**	**32.3**	**27.9**	**17.5**	**6.4** ^c^
Dextran 5000	**5.5**	**3.0**	**2.7** ^c^	1.6	1.2
Dextran 1000	1.3	0.9	0.9	1.3	1.7

FCM, ferric carboxymaltose; FMX, ferumoxytol; IIM, iron isomaltoside 1000; IS, iron sucrose; LMWID, low molecular weight iron dextran; SFG, sodium ferric gluconate. Dextran 50,000 and 5000 served as positive controls and dextran 1000 as a negative control; ^a^ A positive result was defined as A_450_ (sample/blank) ≥2.1 and is shown in bold; ^b^ Starting antibody dilution of 1:1000 was equivalent to 1.0 µg/mL of antibody; ^c^ In bold, the highest dilution of antibody resulting in A_450_ ratio (positive/blank) ≥2.1.

**Table 2:** ELISA results, A_450_ ratios (sample/blank), for the reactivity of IIM and IM1000 (IIM carbohydrate) with 5E7H3 antibody ^a^.

Antibody Dilution	1:1000 ^b^	1:2000	1:4000	1:8000	1:16,000	1:32,000	1:64,000
IIM	**8.6**	**5.1**	**3.5**	**2.2** ^c^	1.4	1.2	1.1
IM1000	0.9	0.9	0.8	0.7	0.7	0.9	0.8
Dextran 5000	**5.2**	**3.9**	**2.9** ^c^	1.9	1.4	1.7	1.2

IIM, iron isomaltoside 1000; IM1000, isomaltoside 1000. Dextran 5000 served as a positive control; ^a^ A positive result was defined as A_450_ (sample/blank) ≥2.1 and is shown in bold; ^b^ Starting antibody dilution of 1:1000 was equivalent to 1.0 µg/mL of antibody; ^c^ In bold, the highest dilution of antibody resulting in A_450_ ratio (positive/blank) ≥2.1.

**Table 3:** Reactivity of IV iron preparations and isomaltoside 1000 with 5E7H3 by reverse single radial immunodiffusion and ELISA.

Assay	Immunodiffusion		ELISA	
	Lot No.	Reaction	Lot No.	Reaction
Dextran 5000	F201	+	00309	+
Dextran 1000	BCBD4347V	−	BCBD4347V	−
FMX	10061002	+	09060402	+
IIM	042838-3	+	949171-1	+
IM1000	n.c.	n.c.	949171-1	−
LMWID	1009019-4	+	1009019-4	+
FCM	144001	−	10667273	−
IS	133001	−	10674663	−
SFG	D7A743A	−	D7A743A	−

FCM, ferric carboxymaltose; FMX, ferumoxytol; IIM, iron isomaltoside 1000; IM1000, isomaltoside 1000; IS, iron sucrose; LMWID, low molecular weight iron dextran; n.c., not conducted; SFG, sodium ferric gluconate.
